#  Hydro-ethanolic Extract of *Portulaca oleracea *Affects Beta-adrenoceptors of Guinea Pig Tracheal Smooth Muscle

**Published:** 2016

**Authors:** Mohammad Hossein Boskabady, Milad Hashemzehi, Mohammad Reza Khazdair, Vahid Reza Askari

**Affiliations:** a *Neurogenic Inflammation Research Centre and Department of Physiology, School of Medicine, Mashhad University of Medical Sciences, Mashhad, Iran. *; b *pharmaceutical Research Center and Department of Physiology School of Medicine, Mashhad University of Medical Sciences, Mashhad, Iran.*; c *Student Research Committee, Faculty of Pharmacy, Mashhad University of MedicalSciences, Mashhad, Iran.*

**Keywords:** *Portulaca oleracea*, Hydroalcoholic extract, β-adrenoceptor, Guinea pig, Tracheal smooth muscle

## Abstract

Thestimulatory effect of the extract of *Portulaca oleracea* (*P. olerace*) on β-adrenoceptor of tracheal smooth muscle was examined.To examine β-adrenoceptor stimulatory effect, concentration response curve to isoprenaline was obtained in pre-contracted tracheal smooth muscle in the presence of three concentrations of aqueous-ethanolic extract, propranolol, and saline. Values of EC_50_ (the effective concentration of isoprenaline, causing 50% of maximum response) and dose ratio-1(CR-1) were measured. This effect was tested innon-incubated tracheal smooth muscle (group 1) and incubated tissues with chlorpheniramine (group 2). Concentration-response curves to isoprenaline in the presence of two higher concentrations of the extract in group 1 and all three concentrations in group 2 showed leftward shifts compared to isoprenaline curves produced in the presence of saline in both groups. EC_50_ obtained in the presence of propranolol was significantly higher than that of saline in both groups of experiments (p<0.05 for both cases). However, the EC_50_ obtained in the presence of two higher concentrations of the extract in group 1 and lower concentration in group 2 were non-significantly but those obtained of medium and high extract concentrations in the group 2 were significantly (p<0.05 for both cases)lower than those of saline. The values of (CR-1) obtained in the presence of all concentrations of the extract in groups1 and 2 were significantly lower than that of propranolol (p<0.05 to p<0.001).The results indicated a stimulatory effect of the *P. olerace *extract on ß_ 2_-adrenoceptors of tracheal smooth muscle.

## Introduction

*Portulaca oleracea *(Purslane) is a grassy plant with small-yellow flowers and height of 10-30 cm, which grows in different areas of the world including north and north-west of Iran. It contains water (92-95%), pectin and lipids (0.3-0.4%), and mucilage (1). Purslane contains more omega-3 fatty acids, alpha-linolenic acid in particular than other leafy vegetable plant. Purslane has 0.01 mg/g of eicosa- pentaenoic acid (EPA) which is an extraordinary amount of EPA for a land-based vegetable source. EPA is an Omega-3 fatty acid found mostly in fish, some algae, and flax seeds. It also contains vitamins (mainly vitamin A, vitamin C, some vitamin B and carotenoids) as well as dietary minerals such as magnesium, calcium, potassium, and iron. It also contains two types of betalain alkaloid pigments, the reddish betacyanins (visible in the coloration of the stems), and the yellow betaxanthins (noticeable in the flowers and in the slight yellowish cast of the leaves). Both of these pigment types are potent antioxidants and have been found to have anti- mutagenic properties (2).

Several therapeutic effects including anti-asthma, anti-inflammatory, antitussive, anti-ascorbic, antipyretic, diuretic and anxiolytic effects were described for *P. olerace*(3-6). This plant was used for antioxidant effect (7-9) and treatment of diabetic patients (10). 

Relaxant effect of the plant on skeletal muscle (11), smooth muscle of the small intestine (12) and its effect on blood pressure (13) were also been demonstrated.The relaxant effect on tracheal smooth muscleforthe plant (14, 15) and its bronchodilatory effect in asthmatic patients (5) as well as an antitussive effect in guinea pigs (3) were shown for the plant in our previous studies.

Therefore, in the present study, the stimulatory effect of *P. olerace* on β-adrenoceptor as the most probable mechanism responsible for is relaxant effect on smooth muscles and its bronchodilatory effect on asthmatic airways was examined. 

## Experimental

*Animals and groups*

Dunkin-Hartley guinea pigsof both sexes (600-800 g) were used in this study. Experiments were performed in compliance with the rulings of the Institute of Laboratory Animals Resources, Commission on Life Sciences (16). Animals were kept in a temperature controlled room with access to standard food and water *ad libitum* and were maintained at 22 ± 2 ºC on a 12 h light/dark cycle during the study period.

Tracheal chains of guinea pigs were prepared as previously described (17) and suspended in a 10 mL organ bath (organ bath 61300, Bio Science Palmer-Washington, Sheerness, Kent U.K.) containing Krebs-Hensele it solution with known composition which was maintained at 37 ^o^C and gassed with 95% O_2_ and 5% CO_2_. Tissue was suspended under isotonic tension (1 g) and allowed to equilibrate for at least 1 h while it was washed with Krebs solution every 15 min. In all experiments contraction or relaxation responses were measured using an isotonic transducer (MLT0202, AD Instruments, Australia) which was connected to a power lab system (PowerLab 8/30, ML870, AD Instruments, Australia). The study was approved by the University›s Ethics Committee. The allowance number of the relevant ethical committee for the animal experiments is 910690.

In order to study the stimulatory effect of *P. olerace* on ß_2_-adrenoceptors, the cumulative log concentration-response curve of isoprenaline sulphate (Sigma Chemical Ltd UK)that induced relaxation of pre-contracted tracheal chains by 10 µM methacholine hydrochloride (Sigma Chemical Ltd UK)was produced as previously described (16). Before producing log concentration-response curve of isoprenaline, tissue were exposed to one of tested solutions for seven minutes included: 10 nM propranolol (0.1 mL of propranolol hydrochloride with 0.1 µM concentration, Sigma Chemical Ltd UK), three concentrations of aqueous-ethanolic extract from *P. olerace* (0.6, 0.12 and 0.25 mg/mL) and 0.2 mL saline. The consecutive concentrations of isoprenaline were added every 2 min (including 5 nM - 1000 µM); and the percentage of relaxation due to each concentration in proportion to the maximum relaxation obtained in the presence of saline was plotted against log concentration of isoprenaline. The effective concentration of isoprenaline causing 50% of maximum response (EC_50_) was calculated as previously described (18).

The slope of the isoprenaline-response curve of each experiment was measured, in order to examine the parallel shift of the curves compared to that of saline. The concentration-ratio minus one (CR-1) as an index of the competitive antagonism was also calculated in experiments with parallel shift in isoprenaline-response curve using the following equation (16):

CR-1= EC50 obtained in the presence of effective solutions EC50 obtained in the presence of saline-1

The study was performed in two different experimental conditionsincluding: 

a) Non incubated tracheal chains (group 1, n = 7).

b) Incubated tracheal chains 30 min prior to the beginning and while obtaining the isoprenaline curve with 1 µM chlorpheniramine maleate (Sigma Chemical Ltd UK), (group 2, n = 5).

All of the experiments were performed randomly with 1 h resting period of tracheal chains between each two experiments while washing the tissues every 15 min with Krebs solution. 

*Plant and extract*

*P. olerace* was purchased from the local market in Mashhad. A voucher specimen was preserved in the Herbarium of the school of pharmacy, Mashhad University of Medical Sciences (Herbarium No: 240-1615-12). The hydro-ethanolic extract was prepared as follows: 100 g of *P. olerace* were grinded and added to 700 mL of ethanol 50% for 72 h at room temperature and the solution was separated by maceration method. This process was repeated for three times. The solutions were dried in room temperature and stored in 4 °C away from light. The plant concentration in the final extract was adjusted to 0.25 g/mL by adding distilled water to the dried extract.

*Statistical analysis*

Data were expressed as mean±SEM. Data of the extract and propranolol were compared with those obtained in the presence of saline using paired t test as well as the comparison of (CR-1) obtained in the presence of extract with those obtained in the presence of propranolol. The values of EC_50_, slope and (CR-1) obtained in two groups were compared using unpaired t test. Significance was accepted at p<0.05.

## Results

C*oncentration-response curves and the values of EC*_50_

**Figure 1:**
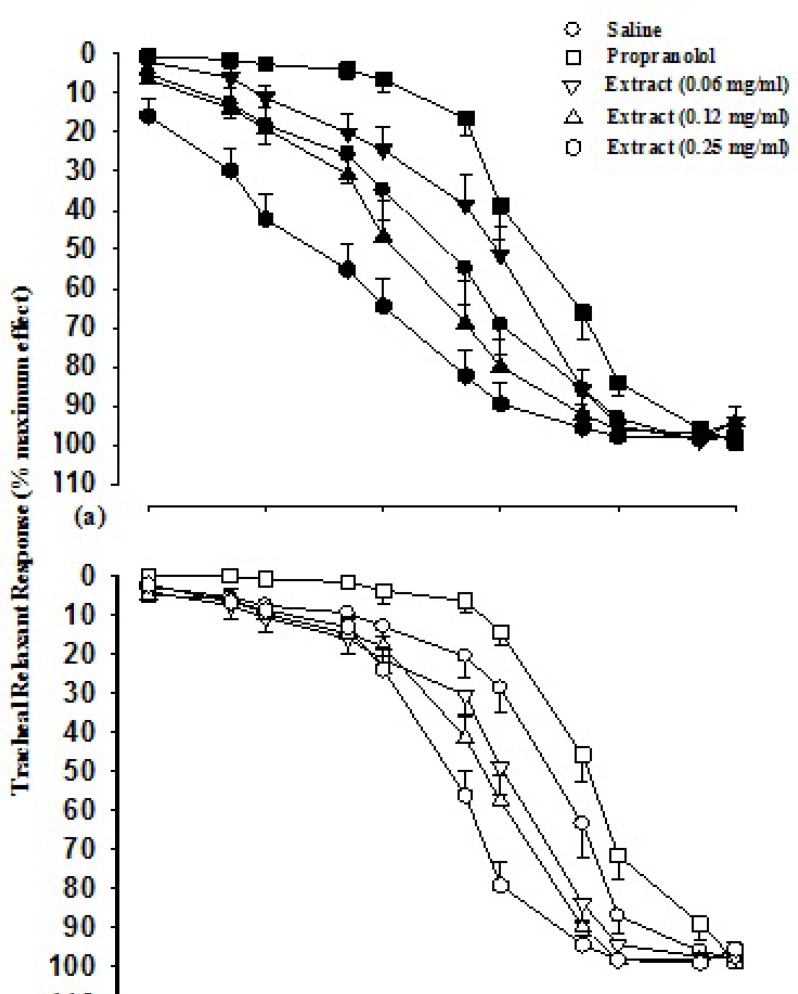
Cumulative log concentration-response curves of isoprenaline induced relaxation of guinea pig tracheal smooth muscle(percent relaxation), in the presence of saline, three concentrations of aqueous-ethanolic extractand 10 nM propranolol. Data are presented as mean±SEM. Group 1 (a) is on non-incubated (n=7), and group 2 (b) on incubated tissues with chlorpheniramine (n=5

Cumulative log concentration-response curves to isoprenaline obtained in the presence of two higher concentrations of the extract in group 1 and all three concentrations in group 2 showed leftward shift while the curve of propranolol showed rightward shift compared to isoprenaline curves produced in the presence of saline in both groups (Figure 1).

**Figure 2:**
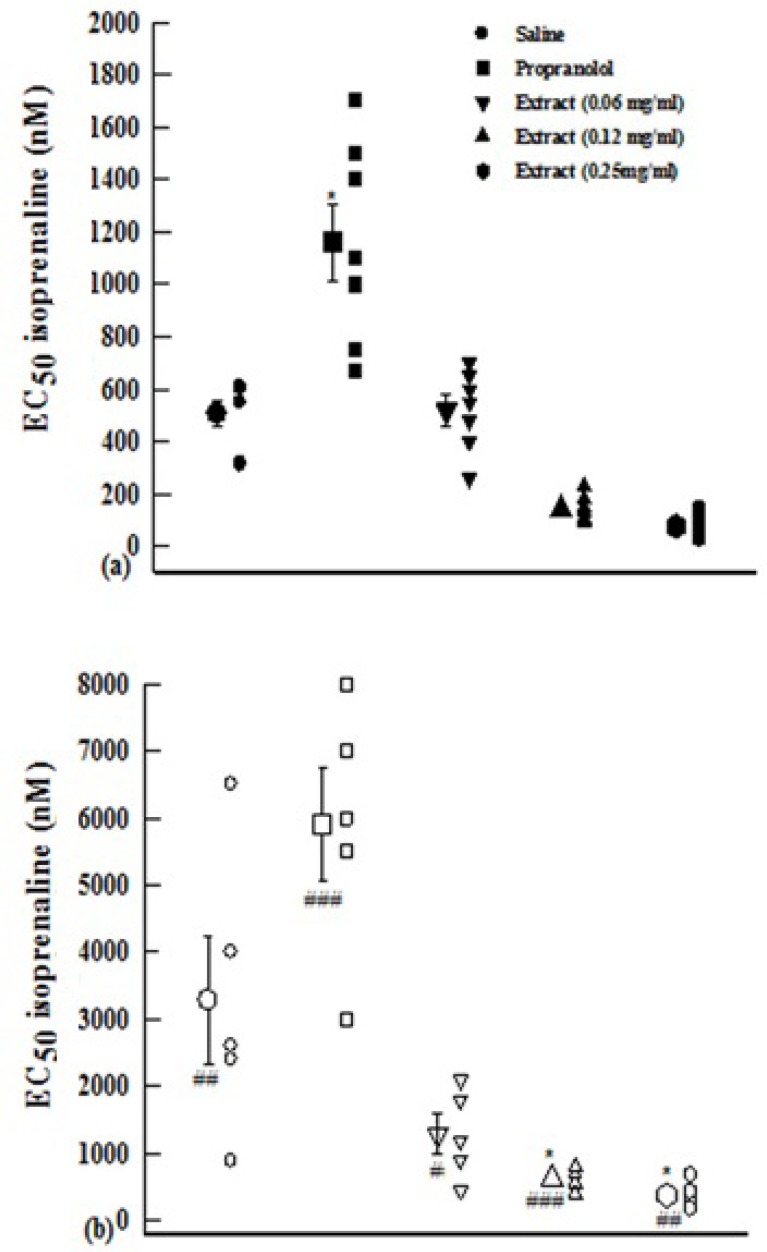
EC_50_ of isoprenaline obtained in the presence of three concentrations of aqueous-ethanolic extract from *P. olerace* (0.06, 0.12, and 0.25 mg/mL), 10 nM propranolol and saline. Data are presented as mean±SEM. Group 1 (a) is on non-incubated (n=7), and group 2 (b) on incubated tissues with chlorpheniramine (n=5).*: p<0.05 compared with saline.#: p<0.05, ##: p<0.01, ###: p<0.001 compared with non incubated tissues

EC_50_ obtained in the presence of propranolol was significantly higher than that of saline in both groups of experiments (p<0.05 for both cases). However, the EC_50_ obtained in the presence of two higher concentrations of the extract in group 1 and lower concentration in group 2 were non-significantly but those obtained in the presence of medium and high extract concentrations in the group 2 were significantly (p<0.05 for both cases) lower than those of saline (Figure 2).

*Shift in isoprenaline concentration-response curves (CR-1)*

**Figure 3.**
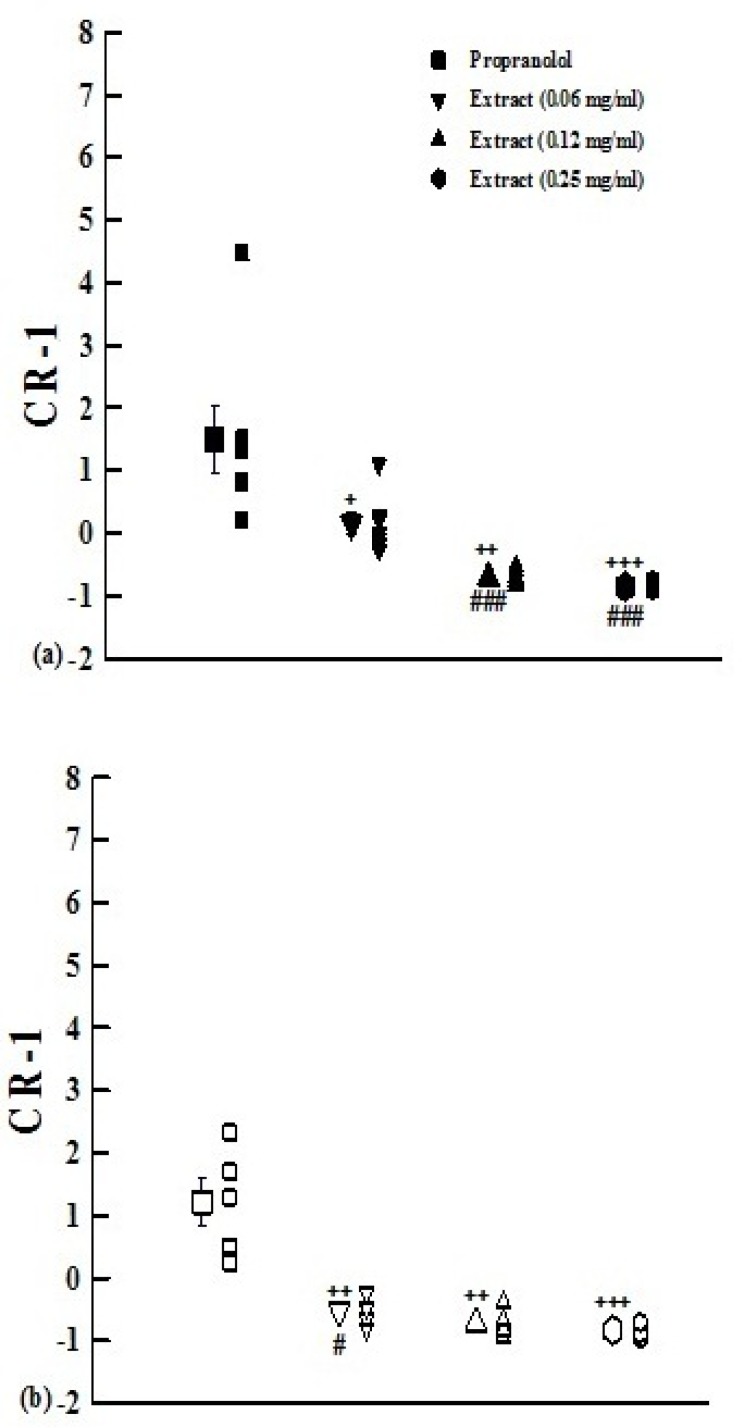
The values of (CR-1) obtained in the presence of three concentrations of aqueous-ethanolic extract from *P.* *olerace*(0.06, 0.12, and 0.25 mg/mL) and 10 nM propranolol.Data are presented as mean±SEM. Group 1 (a) is on non-incubated (n=7), and group 2 (b) on incubated tissues with chlorpheniramine (n=5). +: p<0.05, ++: p<0.01, +++: p<0.001 compared with propranolol.#: p<0.05compared to non incubated tissues

The values of (CR-1) obtained in the presence of medium and high concentrations of the extract in group1 and its all concentrations in group 2 were negative and significantly different from that of propranolol (p<0.05 to p<0.001, Figure 3).

*Slope of isoprenaline-response curves*

The slopes of isoprenaline-response curves obtained in the presence of all three concentrations of the extract were not significantly different from those of saline in both groups (Table 1).

*Correlations between EC*_50_ *and different concentrations of the extract*

There were significant negative correlations between the values of EC_50_ isoprenaline and the extract concentrations in both groups 1 (r = -0.756, p<0.001) and 2 (R = -0.645, p<0.01).

## Discussion

The relaxant effect of *P. olerace* extract on different types of smooth muscles including tracheal smooth muscle has been shown (14, 15).The bronchodilatory effect of this plant extract on asthmatic airways was also documented which was comparable to the effect of theophylline syrup and inhaled salbutamol (5). The most probable mechanism responsible for the relaxant effect of* P. olerace* extract on smooth muscle and its bronchodilatory effect on asthmatic airways is its β-adrenoceptor stimulatory action (19, 20). Therefore, in the present study, the effect of the aqueous-ethanolic extract of the plant on β-adrenoceptor of guinea pig tracheal smooth muscle was examined. For this purpose, cumulative concentration response curve to isoprenaline on precontracted tracheal smooth muscle was performed and the values of EC_50_(The effective concentration of isoprenaline causing 50% of maximum response) and the concentration-ratio minus one (CR-1) were measured.

**Table 1:** Slope of isoprenaline log concentration-response curves in the presence of the extract from *P. olerace*, 10 nM propranolol and saline in two sets of experiments

**olutions**	**Concentration**	**Group 1**	**Group 2**
Saline		0.94±0.02	0.97±0.008
Extract Propranolol	0.06 mg/mL 0.12 mg/mL 0.25 mg/mL	0.887±0.04 0.912±0.02 0.85±0.06 0.91±0.04	0.89±0.02 0.88±0.04 0.96±0.01 0.97±0.007

Data are presented as mean±SEM. Group 1 is on non-incubated (n=7), and group 2 on incubated tissues with chlorpheniramine

(n=5). There were not significant differences between the slopes of concentration response curves obtained in the presence of extract concentrations and atropine with that of saline in either group.

In non-incubated trachea smooth muscle (group 1 experiments), parallel leftward shifts in isoprenaline log concentration-response curves were obtained in the presence of the two higher concentrations of aqueous-ethanolic extract compared to that of saline indicating a possible stimulatory effect ofthe extractonβ-adrenoceptor of guinea pig tracheal smooth muscle (21). The EC_50 _isoprenalineobtained in the presence of two higher concentrations of the extract was non significantly lower than saline but the values of (CR-1) obtained in the presence of all extract concentrations were negative and significantly lower than that of propranolol***. ***These findings indicated a small stimulatory effect of the extract on β-adrenoceptor of tracheal smooth muscle in this group of the experiment (20).

The effect of the plant extract on β-adrenoceptors was also examined in incubated tracheal smooth muscle with chlorpheniramine (group 2 experiments), in order to evaluate the contribution of histamine (H_1_) blocking effect on its stimulatory effect on β-adrenoceptorsseen in group 1***.***

In group 2 there was a parallel leftward shift in isoprenaline-response curves obtained in the presence of all extract concentrations. The EC_50_ isoprenaline obtained in the presence of all extract concentrations in this group were smaller than that of saline which was statistically significant for two higher concentrations. In addition, the values of (CR-1) obtained in the presence of all extract concentration were negative and significantly lower than that of propranolol. The data of group 2 support the stimulatory effect ofthe extract on β-adrenoceptor of tracheal smooth muscle.

The EC_50_ isoprenaline obtained in incubated tissues with chlorpheniramine (group 2) increased relative to those of non-incubated tracheal smooth muscle (group 1). However, there was not statistical difference in the values of (CR-1) between two groups. Therefore, the data of group 2 may also indicate an inhibitory effect for ofextract on histamine (H_1_) receptors.

The results of the present study suggest that the possible mechanism of the relaxant effect of the plant on smooth muscle of small intestine (12), tracheal smooth muscle (14, 15), and its bronchodilatory effect onasthmatic patients (5) areits stimulatory effects on β-adrenoceptors. However, the inhibitory effect of the extract on histamine (H_1_) receptors may also have a small role on its relaxant effect of the plant on smooth muscles.

Our previous study showed relaxant effect of boiled and aqueous extracts of *P. olerace* on tracheal smooth muscle contracted by methacholine or KCl (14, 15). In addition, the relaxant effect of plant extracts on methacholine induced contraction was not significantly different from non-incubated and incubated tissues by propranolol and chlorpheniramine (14, 15). The results of our previous study did not confirm the stimulatory effect of the plant on β-adrenoceptors. Therefore, this effect of the plant was examined more scientifically by producing concentration response curve to isoprenaline in the presence of saline and plant extract. The results showed leftward shift of isoprenaline indicating the stimulatory effect of *P. olerace* extract on β-adrenoceptors.

Therefore, the extracts of *P. olerace* could be of therapeutic value as bronchodilator in obstructive pulmonary diseases. In fact, the bronchodilatory effect of the boiled extract of the plant on asthmatic patients was seen which is may be due to the stimulatory effect of the plant on β-adrenoceptors.

In conclusion, the results of this study suggested a stimulatory effect of *P. olerace *extract on β-adrenoceptors of tracheal smooth muscle. The results also suggested an inhibitory effect of the plant on histamine (H_1_) receptors.
